# Clinical application of multi-parameter ultrasonic assessment for grading rotator cuff injuries: development and validation of a novel scoring system

**DOI:** 10.1186/s12891-026-09487-5

**Published:** 2026-01-07

**Authors:** Xiaona Cai, Chenying Su, Yu Zhan, Rongrong Miao, Chenxian Su

**Affiliations:** 1 Department of Ultrasonography, the First Affiliated Hospital of Wenzhou Medical University, Wenzhou, Zhejiang China; 2 Department of Traditional Chinese Medicine Orthopedics, the First Affiliated Hospital of Wenzhou Medical University, Wenzhou, Zhejiang China; 3 Department of Orthopedics, the First Affiliated Hospital of Wenzhou Medical University, Wenzhou, Zhejiang China

**Keywords:** Multiparameter ultrasonic assessment, Rotator cuff injuries, Novel scoring system

## Abstract

**Background:**

Rotator cuff injuries, a leading cause of shoulder pain and dysfunction, present significant diagnostic challenges. While MRI is the diagnostic gold standard, ultrasonography (US) offers a cost-effective alternative but lacks standardization. This study addresses this gap by developing and validating a novel multiparameter ultrasonic scoring system to increase the diagnostic accuracy and clinical applicability of shoulder ultrasonography for rotator cuff pathology.

**Methods:**

A retrospective diagnostic accuracy study of 252 patients with suspected rotator cuff injury was analyzed. Independent predictors identified through multivariable logistic regression included tear width, tendon swelling, synovial effusion, and vascular score, which were integrated into a weighted composite scoring system. Diagnostic performance was evaluated using receiver operating characteristic (ROC) curve analysis.

**Results:**

The scoring system demonstrated excellent discriminative ability, with an area under the ROC curve (AUC) of 0.92. An optimal diagnostic threshold of > 4 points yielded a sensitivity of 82.2% and a specificity of 94.0%. This system enabled risk stratification into three tiers. Multivariate analysis confirmed tear width, degree of tendon swelling, synovial effusion, and vascular score as key diagnostic indicators.

**Conclusion:**

This ultrasonic scoring system introduces a standardized, quantitative approach to rotator cuff diagnostics, designed to reduce interobserver variability and enhance diagnostic reliability. By stratifying patients into risk categories, it facilitates personalized treatment planning. However, as this represents the initial development and internal validation phase, further prospective and external validation studies are warranted to confirm its broader applicability.

## Background

The shoulder complex, the most mobile joint in the human body, is crucial for daily activities. Despite its wide range of motion, it is highly vulnerable to biomechanical stressors, with rotator cuff pathology being a common musculoskeletal injury.

Epidemiological data shows that rotator cuff injuries are more prevalent in individuals over 40, especially those involved in repetitive overhead work or high-impact sports [1–3]. The clinical consequences of such pathology severely limit functional capacity, as patients often struggle with basic daily tasks like dressing and grooming due to pain-induced weakness. Untreated cases can lead to secondary complications, such as muscle atrophy, joint stiffness, and irreversible degeneration, further reducing quality of life [4, 5].

Current diagnostic protocols for rotator cuff pathology involve evaluating clinical history, symptoms, physical findings, and imaging studies. MRI is the main imaging modality due to its non-invasiveness, superior soft tissue contrast, and multiplanar imaging, which are advantageous for assessing musculotendinous integrity and related abnormalities [6–8]. Shoulder arthroscopy is the gold standard but is invasive and not suitable for initial screening. Conventional radiography and CT have limited value in evaluating soft tissue injuries like rotator cuff tears [9]. While MRI is sensitive and specific for full-thickness tears, it has drawbacks, including high costs, limited accessibility, long scheduling times, and contraindications in some patients.

Ultrasonography offers portability, real-time imaging, and cost-effectiveness. Studies show high diagnostic agreement between preoperative shoulder ultrasonography and arthroscopic findings, especially for full-thickness tears, making it valuable for initial assessment and follow-up. However, there is a lack of unified ultrasound diagnostic standards, often relying on the operator’s experience [10].

Therapeutic strategies for rotator cuff pathology include conservative management and surgery. Nonoperative approaches are for acute cases, partial-thickness tears, or mild impairment, involving activity modification, bracing, medication, and rehabilitation. Surgical intervention is for complete tears, significant deficits, or failed conservative treatment. Arthroscopic repair is now the standard, offering equivalent outcomes with less morbidity than open repair. The goals of surgery are to restore shoulder biomechanics, reestablish musculotendinous continuity, and optimize functional recovery [2, 11].

Current Classification Systems: Rotator cuff pathology lacks standardized nomenclature and diagnostic criteria. Common classifications include: Ellman Classification: Focuses on articular-sided partial-thickness tears; Burkhart Classification: Describes tear patterns based on morphology; Neer Classification: Stages impingement syndrome; Bateman Classification: Categorizes tears by anatomical location; Patte Classification: Grades tendon retraction severity [12–16].

Rationale for Novel Scoring System: These systems have diagnostic variability due to the absence of standardized algorithms and reliance on subjective judgment, leading to interobserver inconsistencies. There are also no quantitative thresholds for surgery or correlations with functional outcomes.

To address these challenges, this study proposes a structured, multiparameter ultrasonic scoring system. By integrating objective ultrasonic parameters with clinical predictors, it aims to:

- Establish standardized diagnostic thresholds for rotator cuff pathology.
- Enable quantitative assessment of tear severity.
- Reduce diagnostic variability with evidence-based criteria.
- Enhance clinical decision-making through prognostic stratification.

This scoring system shifts from descriptive classification to quantitative assessment, with potential implications for treatment algorithm development and comparative effectiveness research in rotator cuff pathology.

## Materials and methods

### Patients and study design

A retrospective diagnostic accuracy study was conducted to evaluate patients with suspected rotator cuff pathology who presented to the author’s hospital between January 2020 and December 2024. The study was derived in a single tertiary centre among patients already suspected of having rotator cuff pathology and considered for surgery. Strict inclusion and exclusion criteria were applied to ensure study homogeneity.

Inclusion Criteria:

1. Clinical suspicion of rotator cuff injury in adult patients (≥ 18 years) confirmed by orthopaedic consultation.
2. Symptoms, including shoulder pain, weakness, or functional limitations.
3. Agreement to undergo standardized diagnostic evaluation protocol.

Exclusion Criteria:

1. Severe systemic comorbidities contraindicating surgical intervention including ASA Class III-IV cardiopulmonary disease, decompensated hepatic/renal failure (Child–Pugh Class B/C or creatinine > 2.5 mg/dL), and active malignancy requiring systemic therapy.
2. Concomitant shoulder pathology including traumatic glenohumeral instability, acute proximal humerus fracture, or a history of ipsilateral shoulder surgery within the preceding 12 months.
3. Local infection such as septic arthritis or osteomyelitis; active cutaneous infection overlying the shoulder girdle.
4. Neuropsychiatric conditions such as uncontrolled seizure disorder, severe dementia (MMSE < 10) or active psychosis.
5. Incomplete medical documentation including the absence of preoperative imaging studies, missing postoperative follow-up data (> 3 months), or inadequate clinical records precluding outcome assessment.
6. Missing data: A total of 25 patients were lost to follow-up and were excluded.

A total of 252 cases were included in this study. Strict application of these criteria ensured that the study population was representative of elective orthopaedic surgical candidates while minimizing confounding variables.

Ultrasound data were collected from eligible patients and subjected to a systematic retrospective analysis, integrating quantitative morphological measurement analysis with semiquantitative scoring of pathological anatomical features. All the imaging data were evaluated in conjunction with the following comprehensive clinical data: (1) patient demographic characteristics, namely, age and sex; (2) clinical manifestations including the presence or absence of restricted shoulder joint mobility((e.g.,The arm-drop test was positive, the Lift-off test was also positive, etc.); the presence or absence of decreased muscle strength (e.g., positive results on the Jobe test, empty-can test, etc.); and the degree of shoulder joint pain; and (3) comorbid rheumatological diseases. All enrolled patients underwent standardized shoulder ultrasonography with systematic documentation of the following parameters: (1) Tendon integrity assessment, including the presence/absence of full-thickness or partial-thickness discontinuity, with quantitative measurements of tear width (in millimetres, mm) when detected; (2) Tendon morphology evaluation, documenting longitudinal/transverse thickness (measured in millimetres, mm, at the thickest point of the rotator cuff tendon with the patient seated and shoulder abducted to 90° and externally rotated) as an indicator of pathological swelling; (3) Synovial analysis quantifying the maximum depth of joint effusion (in millimetres, mm, measured at the deepest point of fluid accumulation within the joint space) within the subacromial-subdeltoid bursa; (4) Vascularity assessment using Power Doppler ultrasound, with semiquantitative grading of intratendinous/peritendinous blood flow distribution: Normal perfusion (0) / Hypo- or hypervascularity (1), where changes in vascularity may indicate inflammation or a healing response; (5) Osseous evaluation for heterotopic calcification (hyperechoic foci with posterior acoustic shadowing). Sonographers were blinded to clinical, MRI, and arthroscopic findings to minimize bias. And one has to admit, the limitations of operator dependency in ultrasound interpretation.

**Fig. 1:**
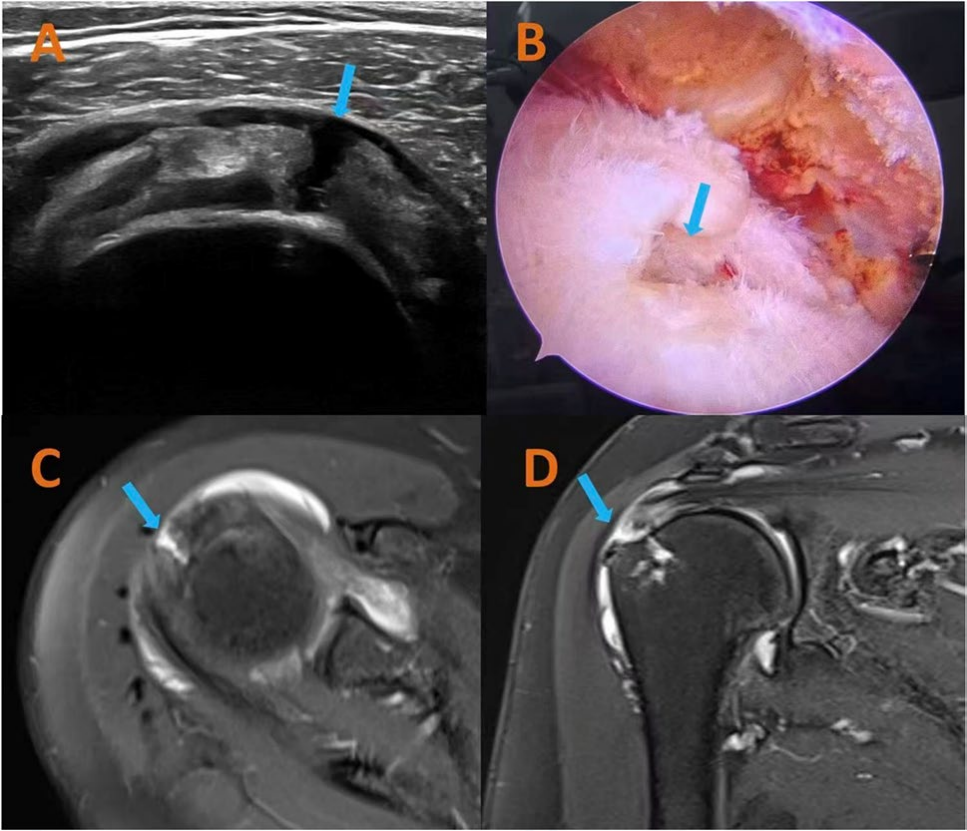
Ultrasound, magnetic resonance imaging (MRI) images, and intraoperative photographs of typical rotator cuff tendon tears. **A** Ultrasound image of a full-thickness tear of the rotator cuff. The arrow indicates the site of the tendon tear. **B** Intraoperative images of the patient during the arthroscopic surgery. The arrow indicates the site of the tendon tear. **C** Magnetic resonance transverse image of full-thickness tear of the rotator cuff. The arrow indicates the site of the tendon tear. **D** Magnetic resonance coronal image of full-thickness tear of the rotator cuff. The arrow indicates the site of the tendon tear

Adjunctive diagnostic evaluations included (1) MRI with dedicated shoulder coil protocols (1.5T/3T systems, T1/T2-weighted sequences, fat suppression techniques) and (2) serological analysis comprising complete blood count with differential, C-reactive protein, erythrocyte sedimentation rate, and a comprehensive metabolic panel (including hepatic/renal function biomarkers). Figure 1 presents typical ultrasound images, MRI images, and intraoperative photos of typical rotator cuff tendon tears.

The diagnostic ultrasound systems used in this study included the Hitachi HI VISION Avius paired with a 10 − 2 MHz broadband linear transducer, the Mindray Resona 8Pro equipped with a high-frequency linear array transducer, and the GE Voluson E8 utilizing a multifrequency convex transducer, ensuring comprehensive imaging capabilities across diverse clinical applications.

### Statistical analyses

The scores were analysed by ROC curve analysis, and sensitivity and specificity were calculated with SPSS 24.0 software. Pearson’s chi-square or Fisher’s exact test was used to compare baseline characteristics. Logistic regression analysis was used to identify independent predictors of rotator cuff injury and to establish a risk assessment model. The diagnostic performance of the scoring system was evaluated through ROC curve analysis, with AUC calculations and optimal cut-off determination using the Youden index. The sensitivity, specificity, positive predictive value (PPV), and negative predictive value (NPV) were computed with 95% confidence intervals (CIs). Tests were two-sided. A p value < 0.05 was considered to indicate statistical significance, and robust estimates of the standard error were used in all regression analyses.

#### Baseline comparisons

Categorical variables were compared using Pearson’s chi-square test or Fisher’s exact test (when cell counts < 5 were present). Continuous variables were analysed via Student’s t test for normally distributed data or the Mann–Whitney U test for nonparametric distributions following the Shapiro–Wilk normality assessment.

#### Predictive modelling

Multivariate logistic regression analysis with backwards stepwise selection (p-entry = 0.05, p-removal = 0.10) was employed to identify independent predictors of symptomatic rotator cuff pathology.

## Results

### Patient cohort characteristics

A total of 252 patients meeting the inclusion criteria underwent definitive diagnostic evaluation for rotator cuff pathology. In this study, we strictly adhered to uniform standards to define positive and negative groups. The positive group was determined based on either the discovery of distinct pathological features through MRI or direct confirmation of lesions during surgical procedures (including arthroscopy and other relevant surgeries). The negative group was established after a comprehensive clinical assessment, which included a detailed medical history inquiry and physical examination, along with no obvious pathological features detected by MRI. Moreover, patients in this group did not exhibit any worsening of symptoms or signs related to the target disease during subsequent follow-up visits. Patients were categorized into positive (*n* = 160, 63.5%) and negative (*n* = 92, 36.5%) diagnostic groups on the basis of imaging/arthroscopic findings, with 145 patients (90.6%) in the positive group subsequently undergoing arthroscopic intervention.

### Demographic distribution

The positive group included 67 males (41.9%) and 93 females (58.1%), whereas the negative group included 42 males (45.7%) and 50 females (54.3%). The age distributions of the cohorts were comparable, with the positive group having a mean age of 66.79 ± 8.32 years (median 68 years) and the negative group having a mean age of 66.77 ± 7.94 years (median 69 years). The chi-square test was used for p values > 0.05, and there were no significant differences between the two groups in terms of age or sex. A logistic analysis of the factors influencing rotator cuff injury is shown in Table 1.

**Table 1:** Logistics analysis of influencing factors of rotator cuff injury

Variable	B	Std. Error	Wald	df	Significance	Exp(B)	95% CI Lower	95% CI Upper
Gender (Female = 0, Male = 1)	-0.939	0.478	3.863	1	*0.049*	0.391	0.153	0.997
Age	-0.023	0.021	1.254	1	0.263	0.977	0.939	1.017
Restricted Movement	2.324	0.529	19.295	1	*< 0.001*	10.212	3.621	28.799
Muscle weakness	1.772	0.502	12.474	1	*< 0.001*	5.883	2.201	15.728
Pain	2.478	0.537	21.278	1	*< 0.001*	11.922	4.159	34.173
Comorbid Rheumatoid Arthritis	1.345	0.481	7.800	1	*0.005*	3.836	1.493	9.856
Tear width	1.342	0.326	16.895	1	*< 0.001*	3.826	2.018	7.256
Tendon Thickness	0.723	0.135	28.760	1	*< 0.001*	2.061	1.582	2.684
Joint Effusion	0.601	0.123	23.994	1	*< 0.001*	1.825	1.434	2.321
Calcification	0.939	0.541	3.009	1	0.083	2.557	0.885	7.388
Vascularity Score	2.238	0.546	16.771	1	*< 0.001*	9.370	3.211	27.341
Constant	-7.120	1.795	15.730	1	*< 0.001*	0.001		

Exp(B) represents the odds ratio for each independent variable; 95% CI = 95% confidence interval for Exp(B); Variables with *p* < 0.05 were considered statistically significant predictors

Multivariate analysis confirmed significant associations between rotator cuff injury and female sex, restricted mobility, muscle weakness, shoulder pain, comorbid rheumatoid arthritis, increased tear width, tendon hypertrophy, joint effusion, and abnormal vascularity scoring (all *p* < 0.05). These factors constituted independent predictors incorporated into a risk assessment model demonstrating strong discriminative ability (C-statistic = 0.81) and calibration (Hosmer–Lemeshow *p* = 0.76) and were used to construct a clinical risk layer to guide treatment decisions. Ultrasound parameters, including tear dimensions, effusion depth, and vascularity scoring, showed diagnostic specificity, while calcification demonstrated limited utility and was more strongly correlated with calcific tendinitis than with rotator cuff pathology. The final predictive algorithm, validated through associations between elevated vascularity scores and postoperative complications, provides a quantitative framework for personalized risk evaluation in shoulder pathology. On the basis of the logistic analysis results, we established a risk model: Risk Score = − 7.12 − 0.939×Sex (Male = 1) + 2.324×Restricted Mobility (Yes = 1) + 1.772×Muscle Weakness (Yes = 1) + 2.478×Pain (Yes = 1) + 1.345×RA (Yes = 1) + 1.342×Tear width + 0.723 × Tendon Thickness + 0.601×Joint Effusion + 2.238×Vascularity Score.

To standardize diagnostic protocols and mitigate interobserver variability, we developed a structured scoring system (Table 2) that integrates clinical manifestations and ultrasonographic parameters derived from multivariable logistic regression analysis. This algorithm systematizes diagnostic evaluation for suspected rotator cuff injuries, enhancing diagnostic consistency across institutions and specialties.

**Table 2:** Composite diagnostic scoring system integrating clinical and ultrasonographic parameters

Scoring Domain	Parameters	Scoring Criteria	Points
Clinical Manifestations			
1	Shoulder range of motion limitation	Absent (0) / Present (1)	0–1
2	Muscle weakness	Absent (0) / Present (2)	0–2
3	Shoulder pain	Absent (0) / Present (1)	0–1
4	Comorbid rheumatoid arthritis	Absent (0) / Present (1)	0–1
Ultrasonographic Parameters			
5	Tear width	Intact (0) / <30 mm (1) / 30–50 mm (2) / >50 mm OR complete rupture (3)	0–3
6	Tendon thickness	Normal (0) / 1–5 mm swelling (1) / ≥6 mm swelling (2)	0–2
7	Joint effusion depth	<3 mm (0) / ≥3 mm (1)	0–1
8	Vascularity (CDFI assessment)	Normal perfusion (0) / Hypo- or hypervascularity (1)	0–1
Total Score		Composite score range: 0–12	

*CDFI* colour Doppler flow imaging

### Validation and clinical application

**Fig. 2:**
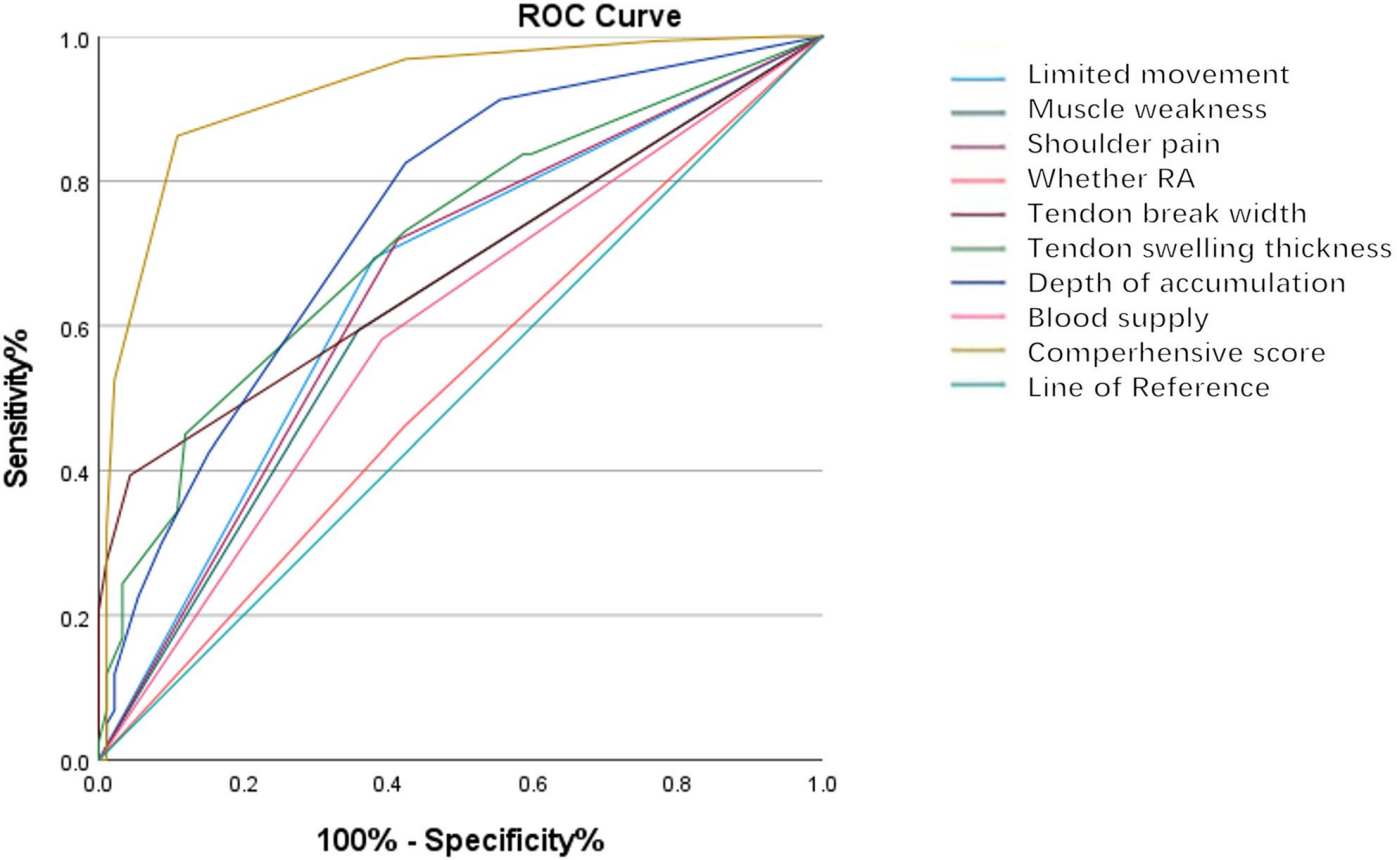
Comparative ROC analysis of multivariate diagnostic indicators for rotator cuff injury: Validation of a comprehensive scoring system. ROC curve analysis demonstrated excellent diagnostic performance, with an AUC of 0.923 (95% CI 0.887–0.959). ROC: receiver operating characteristic; AUC: area under the ROC curve; RA: rheumatoid arthritis.

ROC curve analysis demonstrated excellent diagnostic performance, with an AUC of 0.923 (95% CI 0.887–0.959), as shown in Fig. 2. The area under the curve (AUC) for activity restriction is 0.657, with a 95% confidence interval ranging from 0.586 to 0.728. For swelling, the AUC is 0.618, with a confidence interval of 0.546 to 0.689. Pain demonstrates an AUC of 0.653, with a confidence interval spanning from 0.581 to 0.724. Comorbid rheumatoid arthritis has an AUC of 0.519, and its confidence interval is 0.445 to 0.593. The AUC for tear width is 0.680, with a confidence interval of 0.616 to 0.744. Tendon swelling shows an AUC of 0.717, and its confidence interval ranges from 0.653 to 0.780. Joint effusion has the highest AUC among individual variables at 0.748, with a confidence interval of 0.684 to 0.812. The vascularity score has an AUC of 0.595, and its confidence interval is 0.522 to 0.668. Finally, the comprehensive score exhibits an outstanding AUC of 0.923, with a very narrow confidence interval of 0.887 to 0.958. The optimal diagnostic threshold was established at > 4 points. The sensitivity was 82.2% (95% CI 74.3–88.5%), and the specificity was 94.0% (95% CI 89.2–97.1%).

**Fig. 3:**
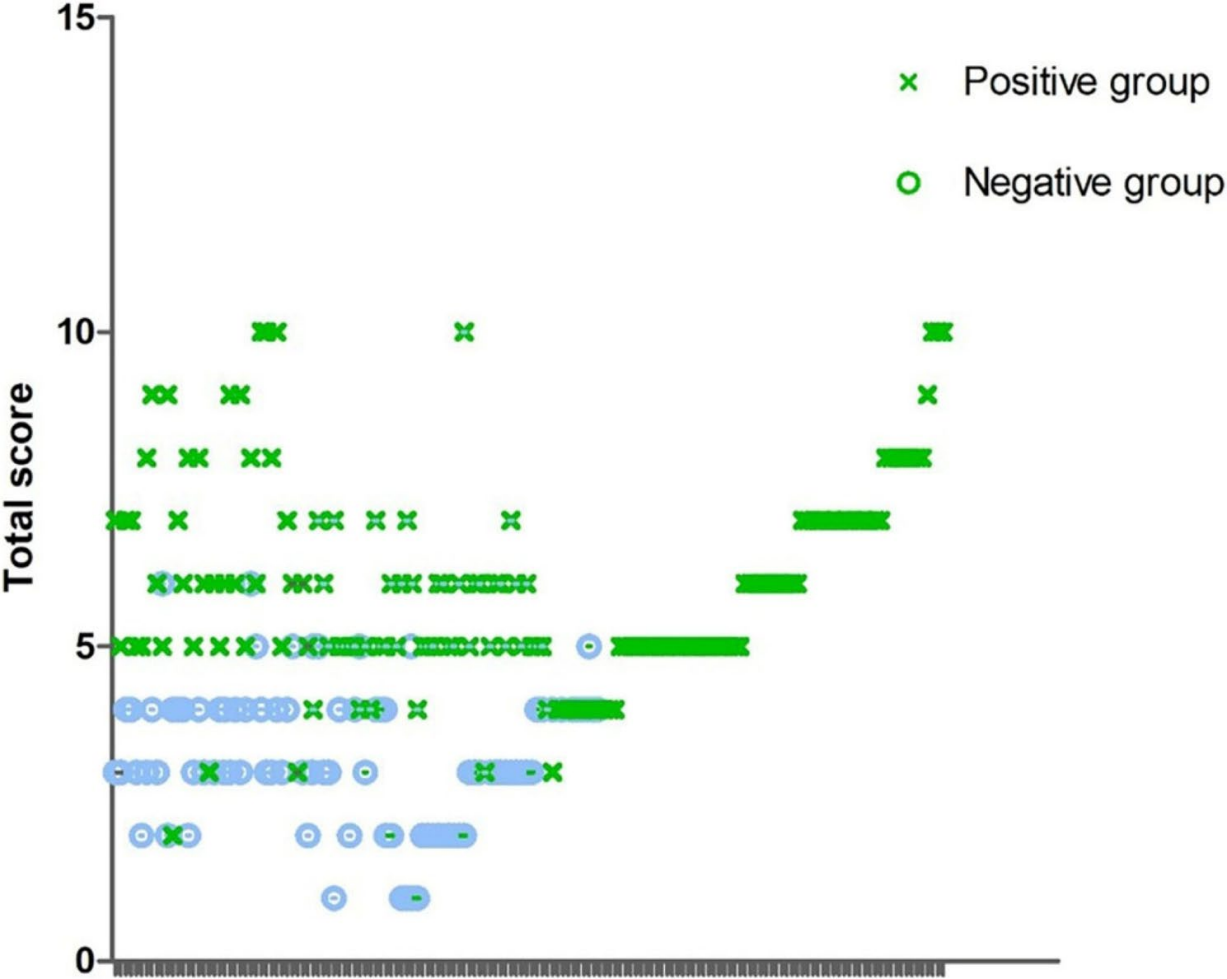
Scatter plot showing the distribution of scores between the positive group and the negative group. A total scores > 4 indicates a high probability of rotator cuff injury. In the group with scores > 4, there were a total of 194 cases, among which 175 were positive cases, and the PPV was 90.2%. In the group with scores ≤ 4, which consisted of 58 cases, the NPV was 91.4%.

Scatterplot visualization (Fig. 3) confirmed the bimodal score distribution between the confirmed injury (positive group) and normal (negative group) cohorts. Following multidisciplinary consensus with orthopaedic surgeons and radiologists, we recommend the following:

- *Diagnostic Threshold*: A total scores > 4 indicates a high probability of rotator cuff injury. In the group with scores > 4, there were a total of 194 cases, among which 175 were positive cases, and the PPV was 90.2%. In the group with scores ≤ 4, which consisted of 58 cases, the NPV was 91.4%.
- *Clinical Correlation*: Elevated scores correlate with injury severity, necessitating comprehensive evaluation, including MRI, for surgical planning.

Clinically, the model facilitates three-tier risk stratification:

- Low - risk cohort (0–4 points): This group is considered to have a relatively low probability of significant rotator cuff pathology. Patients in this category are suitable for conservative management and regular follow - up. Rescoring should be carried out if the symptoms worsen.
- Moderate - risk group (5–7 points): Patients in this group have an intermediate risk of rotator cuff injury, especially partial - thickness tears. MRI correlation is needed to further evaluate the extent of the tear prior to finalizing surgical planning.
- High - risk category (≥ 8 points): The probability of irreparable tearing is significantly increased in this group. Preoperative biomechanical assessment and planning of tendon transfer are necessary.

### Clinical significance

This scoring system provides a quantifiable framework for the following:

1. Standardizing diagnostic workflows across health care settings.
2. Facilitating objective communication between clinicians and sonographers.
3. Reducing diagnostic discrepancies through structured parameter assessment.
4. Guiding triage decisions based on validated risk stratification.

Compared with conventional protocols, the implementation of this tool demonstrates improved diagnostic accuracy, particularly in equivocal cases where clinical suspicion and imaging findings require systematic integration.

## Discussion

Rotator cuff injuries are a common musculoskeletal condition with substantial clinical and socioeconomic impacts. While MRI and arthroscopy remain the gold standards for diagnosis, each has significant drawbacks: MRI, despite its superior soft-tissue contrast, is costly, involves long wait times, and is contraindicated in patients with claustrophobia or implantable devices, while motion artifacts in elderly patients due to poor compliance further reduce its diagnostic accuracy; arthroscopy, though offering therapeutic benefits, is invasive and carries risks such as infection, iatrogenic cartilage damage, and anesthesia-related complications, limiting its use to specific cases.

Emerging evidence supports the integration of ultrasonography as a viable diagnostic alternative with demonstrated performance comparable to that of established modalities [17–19]. A prospective investigation by Behzad Aminzadeh et al. (2018) [20] rigorously assessed diagnostic accuracy in 48 patients with clinically suspected rotator cuff pathology. The researchers performed shoulder ultrasonography and concurrent MRI (serving as the reference standard) on all the participants. Diagnostic performance metrics, including specificity, sensitivity, and positive/negative predictive values, were calculated for ultrasonographic detection of rotator cuff disorders.

Among the diagnosed conditions, partial-thickness tears constituted the largest subgroup (17 cases, 35.4%), followed by full-thickness tears (16 cases, 33.3%) and tendinopathy (10 cases, 20.8%). Notably, ultrasonography demonstrated exceptional diagnostic performance: 93.7% accuracy for full-thickness tears, 90% sensitivity for tendinopathy, and 100% specificity for full-thickness tears. The modality also achieved 96.7% specificity for partial-thickness tears, underscoring its reliability in distinguishing tear morphology [20].

These findings underscore the diagnostic precision of ultrasonography across the spectrum of rotator cuff pathologies, particularly in detecting structural discontinuities. Notably, the perfect specificity (100%) for full-thickness tears establishes ultrasonography as a reliable screening tool to rule out irreparable defects. Complementary validation through Miguel Jimenez-Vasquez et al.’s (2020) [9] cohort study further corroborates these results. In 37 patients who underwent preoperative ultrasonography followed by diagnostic arthroscopy, the two modalities demonstrated substantial diagnostic concordance (Cohen’s κ = 0.76, *p* < 0.001), with an overall agreement rate of 81%. This consistency persists despite the inherent limitations of arthroscopy as a purely intra-articular assessment tool, which may lead to the underestimation of extracapsular pathologies detectable via ultrasonography [9]. Ultrasound provides clear visualization of muscles, tendons, ligaments, peripheral nerves, and their anatomical relationships, enabling precise assessment of lesion scope and severity. Its simple operation and low cost make it highly suitable for widespread use in primary healthcare settings. Ultrasonography has emerged as an indispensable diagnostic tool in modern musculoskeletal practice, offering a favourable balance of diagnostic performance, cost efficiency, and patient acceptability. Its integration with clinical scoring systems represents a new method towards personalized, evidence-based care pathways for rotator cuff pathology [21, 22]. Based on the existing studies mentioned above, since ultrasound has the aforementioned advantages, this research proposes a new diagnostic method by integrating clinical parameters and ultrasound parameters, providing a new diagnostic approach.

Despite advancements in musculoskeletal imaging, current ultrasonographic diagnosis of rotator cuff pathology remains in its developmental infancy. Diagnostic standardization is suboptimal, with marked variability in procedural protocols and interpretive criteria across institutions. This diagnostic ambiguity stems from the reliance on operator-dependent qualitative assessments rather than quantifiable scoring systems, thereby limiting reproducibility and diagnostic confidence.

The heterogeneity in rotator cuff injury classification further complicates clinical communication and research comparability. Five predominant classification systems are currently utilized, each with distinct methodological foundations and clinical applications:

Several classification systems for rotator cuff tears have been developed. The Ellman Classification [12] is an arthroscopic system that differentiates partial-thickness tears (subcategorized into articular-sided, bursal-sided, and intratendinous with depth stratification) and full-thickness tears (anatomically designated by tendon and size stratified, with a defect area calculation method). The Burkhart Classification (2006) [13, 14] is a histopathological staging system based on progressive tendon degeneration, with three stages from tendinitis to complete full-thickness rupture. The Bateman Classification (1965) [15] is an anatomic-morphological grading of full-thickness tears after stump debridement, with four grades based on diameter. The Patte Classification [16] is a retraction-based grading system for displaced tendon ends, with three grades according to the degree of retraction.

The established efficacy of structured ultrasonographic scoring systems in thyroid and breast nodule assessment underscores the translational potential of similar methodologies for musculoskeletal pathologies. Given the diagnostic heterogeneity inherent to rotator cuff injury evaluation, the development of a standardized ultrasound-based scoring protocol incorporating quantitative parameters and validated clinical correlates represents a critical unmet need in sports medicine and orthopaedic practice.

Beyond imaging, clinical symptoms are indispensable for diagnosing rotator cuff injuries, as pain severity and functional impairment strongly correlate with histopathological tendon degeneration, while contemporary rheumatology research increasingly recognizes the pathophysiological link between RA and rotator cuff pathology, wherein chronic RA synovitis drives tendon degeneration through multiple mechanisms: synovial hyperplasia-induced pannus formation at the rotator interval causes mechanical friction and vascular compromise, accelerating tendon fraying; elevated matrix metalloproteinase (MMP)-3 and MMP-9 activity in RA synovial fluid disrupts collagen homeostasis, weakening tendon biomechanics; chronic inflammation exacerbates supraspinatus muscle atrophy, with MRI revealing 37% greater fatty infiltration in RA patients compared to age-matched controls; and long-term glucocorticoid use in RA management impairs tendon cell viability and collagen synthesis, further contributing to tendinopathy.

These mechanisms collectively render RA patients 2.8-fold more susceptible to full-thickness rotator cuff tears than the general population is (*p* = 0.001). Recognizing this clinical association, the current diagnostic protocol incorporates RA status as a binary covariate (present/absent) in the multivariable scoring algorithm, with a weighted coefficient of β = 1.34 (95% CI 1.02–1.66; *p* = 0.002). This adjustment aligns with emerging paradigms in musculoskeletal ultrasonography that emphasize comorbidity-stratified diagnostic thresholds [23, 24].

This study improved ultrasonography’s diagnostic accuracy for rotator cuff injuries via standardized protocols and quantitative assessment. Multivariate analysis identified eight predictors, which informed the development of a risk-stratified management system. This integrates histopathologically validated RA mechanisms and objective thresholds for tendon parameters. Enabling personalized treatment and supporting longitudinal validation, with future plans to enhance diagnostic precision through the incorporation of shear-wave elastography.

There are several limitations to this study. First, the retrospective single - centre design introduces selection bias and limits generalizability. And the inter-observer and intra-observer reliability analyses of the key ultrasound parameters were lacking. Second, while the cohort size (*n* = 327) exceeds that of prior studies, the relatively small sample precludes robust evaluation of rare events and low - prevalence covariates (e.g., collagenopathies and iatrogenic injuries). Since the analysis combines these surgical cases with those confirmed only by MRI, there is a potential for verification bias. Third, unmeasured confounders, including occupational factors and genetic predisposition, could not be assessed because of retrospective data limitations. Moreover, due to the retrospective design and constraints in sample size, formal internal validation procedures such as bootstrapping or cross - validation were not feasible in this study. As a result, the reported performance metrics, including AUCs, may be subject to overfitting, and the findings should be interpreted as preliminary. Multi - institutional prospective studies with standardized protocols and longitudinal follow - up are needed to validate these findings, clarify the natural disease history, and assess the generalizability of the results. Large prospective studies are also crucial to confirm these findings and further elucidate the natural history of this disease.

## Conclusion

This study proposes a nascent structured ultrasonographic scoring system that might enhance the diagnostic precision in the evaluation of rotator cuff injuries. By incorporating quantitative tendon parameters, clinical symptoms, and the co - occurrence of rheumatoid arthritis, our preliminary analysis yielded an 82.2% sensitivity and 94.0% specificity, along with a ROC AUC of 0.923. These early results hint at the potential superiority of our approach over conventional qualitative methods, but conclusive evidence requires further investigation. The innovation lies in three domains: (1) Initiate the development of a validated risk stratification model incorporating eight independent predictors (limited active range of motion, muscle weakness, resting pain severity, rheumatoid arthritis history, tendon thickness, tear retraction depth, shoulder joint effusion, and vascularity grade); (2) the establishment of evidence-based diagnostic thresholds; and (3) the creation of a tiered management algorithm that reduces unnecessary imaging while identifying high-risk patients requiring surgical intervention. This multidimensional approach not only enhances diagnostic accuracy but also facilitates personalized treatment planning. However, as this represents the initial development and internal validation phase, a multi-center and further prospective and external validation studies are warranted to confirm its broader applicability.

## Data Availability

The datasets used and analysed during the current study are available from the corresponding author on reasonable request.

## Abbreviations

MRI: Magnetic resonance imaging
ROC: Receiver operating characteristic
AUC: Area under the ROC curve
CT: Computed tomography
IRB: Review Board
CDFI: Colour Doppler flow imaging
PPV: Positive predictive value
NPV: Negative predictive value
CIs: Confidence intervals
RA: Rheumatoid arthritis
MMP: Matrix metalloproteinase
